# Supplementary material for: Computational Screening of All Stoichiometric Inorganic Materials
Source: Chem. 2016 Oct 13;1(4):617–27. doi: 10.1016/j.chempr.2016.09.010 (PMC5074417; doi:10.1016/j.chempr.2016.09.010)
Supplement: Document S1. Supplemental Experimental Procedures, Figures S1 and S2, and Tables S1 and S2 [file mmc1.pdf]

Chem, Volume *1*

Supplemental Information

Computational Screening of All Stoichiometric Inorganic Materials

Daniel W. Davies, Keith T. Butler, Adam J. Jackson, Andrew Morris, Jarvist M. Frost, Jonathan M. Skelton, and Aron Walsh

I. SUPPLEMENTAL DATA ITEMS

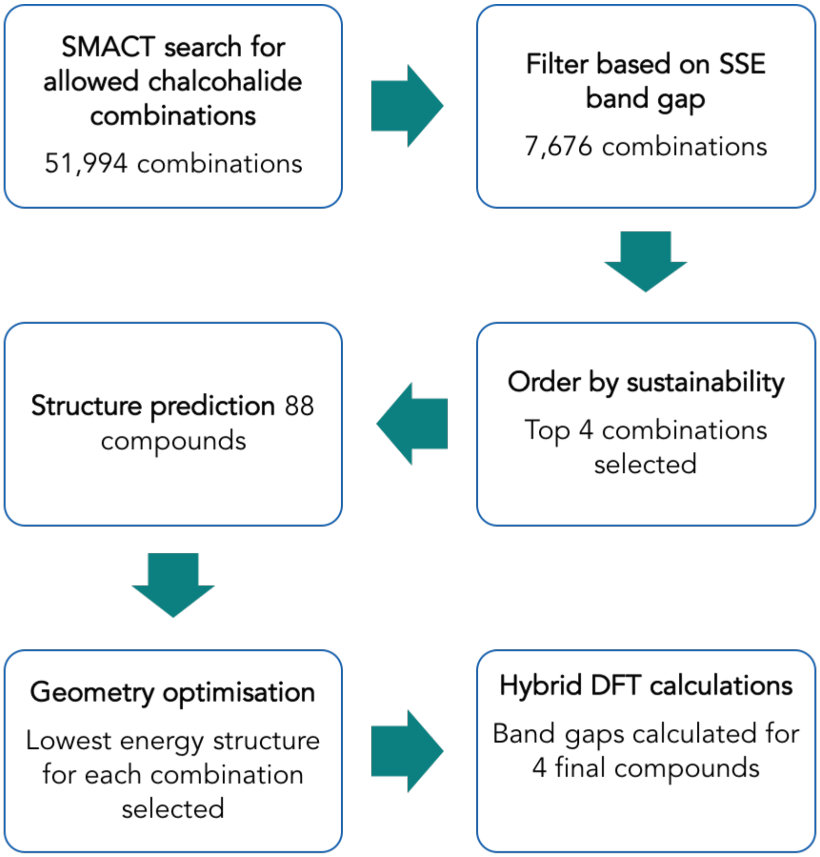

FIG. S1. Computational workflow: searching the combinatorial space for photoelectrochemical water splitting materials.

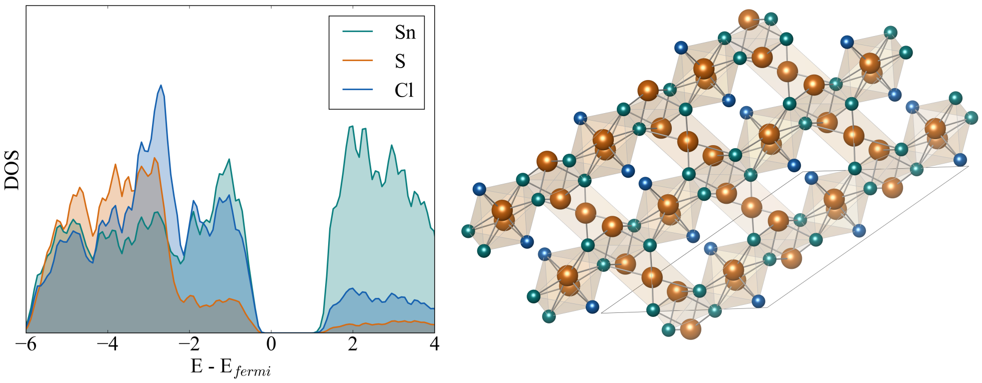

FIG. S2. (left) Electronic density of states and (right) predicted crystal structure of $Sn_5S_4Cl_2$.

TABLE S1: Comparison of measured bandgaps[1] (E_g^{exp}) against those predicted from the SSE model (E_g^{SSE}).

Material	E_g^{exp} (eV)	E_g^{SSE} (eV)
$MgSiP_2$	2.60	2.03
$ZnSiP_2$	1.70	2.00
$ZnSiAs_2$	1.00	1.93
$ZnGeN_2$	4.00	2.67
$ZnGeP_2$	1.70	2.14
$ZnGeAs_2$	1.00	1.15
$ZnSnP_2$	1.30	1.66
$ZnSnAs_2$	0.60	0.75
$ZnSnSb_2$	0.50	0.40
$CdSiP_2$	1.20	2.20
$CdSiAs_2$	0.50	1.55
$CdGeP_2$	1.20	1.73
$CdGeAs_2$	0.50	0.57
$CdSnP_2$	1.20	1.17
$CdSnAs_2$	0.50	0.26
$ZnGa_2S_4$	2.40	3.25
$ZnGa_2Se_4$	2.60	2.18
$ZnIn_2S_4$	1.80	2.87
$ZnIn_2Se_4$	2.00	1.68
$ZnIn_2Te_4$	1.40	1.35
$CdAl_2S_4$	1.90	3.40
$CdGa_2S_4$	1.90	3.16
$CdGa_2Se_4$	2.10	2.33
$CdGa_2Te_4$	1.50	1.50
$CdIn_2S_4$	1.80	2.21
$CdIn_2Se_4$	2.00	1.83
$CdIn_2Te_4$	1.40	1.15

$MgGa_2S_4$	2.50	3.40
$MgGa_2Se_4$	2.70	2.20
AsSBr	1.40	2.50
SbSI	1.50	1.88
SbSBr	1.50	2.26
SbSeBr	1.70	1.92
SbSeI	1.50	1.68
SbTeI	1.10	1.28

TABLE S2: Calculated bandgaps of top compounds identified by the screening procedure based upon density functional theory calculations (HSE06 functional) of the predicted crystal structures.

ABC combination	Formula	E_g^{calc} (eV)
CdSCl	$Cd_5S_4Cl_2$	2.96
CdSF	Cd_4SF_6	3.40
SnSCl	$Sn_5S_4Cl_2$	1.62
SnSF	Sn_4SF_6	3.00

II. SUPPLEMENTAL COMPUTATIONAL PROCEDURES

A. Validation of ternary bandgaps using the solid-state energy scale

The SSE dataset was initially built from binary compounds. In the original paper[2] the authors speculate about its applicability to ternary and higher order materials; however, we can find no reports of any such application. In order to assess whether the bandgap of a ternary material can be estimated from the difference between the highest anion and lowest cation SSE, we have tested this method against a set of well-characterised ternary semiconductor bandgaps.[1]. We compare to 35 materials, covering III-IV-V_2, II-III_2-VI_4 and V-VI-VII compounds, including metal halides, chalcogenides and pnictides. The agreement is reasonable, with a root-mean-squared deviation between of 0.66 eV. The data are presented in Table S1.

B. Workflow for selecting candidate photoelectrodes

The six step procedure that we adopt is shown schematically in Figure S1.

1. *Allowed chalcohalide combinations*

The constraints of charge neutrality and electronegativity are applied to all possible $A_xB_yC_z$ combinations with $B = [O, S, Se, Te]$ and $C = [F, Cl, Br, I]$. Stoichiometry is restricted to $A_xB_yC_z$, where the integers $w, x, y, z \leq 8$. Additionally we limit the A cations to those with an SSE higher than the water reduction potential (approx. -4.5 V relative to the vacuum at pH = 0). This results in 51,994 combinations.

2. *SSE bandgap filter*

The elemental combinations with a bandgap outside the range of 1.5 – 2.5 eV according to the SSE scale are discarded. Since $\sim$ 2 eV would represent an ideal bandgap, the $\pm$0.5 eV range allows sufficient space to allow for the uncertainty in the predicted SSE values. This results in 7,676 allowed combinations.

3. Sustainability filter

The sustainability of the 7,676 $A_xB_yC_z$ combinations is assessed based on sum the HHI_R scores of the three elements. The 20 combinations with the smallest HHI_R scores are shown in Figure 2 and the four combinations with the smallest HHI_R scores are taken forward to the structure prediction stage.

4. Structure prediction

In order to ascribe three-dimensional structures to the four element combinations, we use the approach developed by Hautier *et al.*[3] based on structural analogy. It suggests probable structure types based on the likelihood of ionic substitutions in existing compounds with known crystal structures. This procedure enables a rapid screening step which returns possible compounds with an associated probability of each crystal structure being adopted. We use a probability threshold of 0.001 and the Materials Project as the database for existing compounds. This results in a total of 88 structures to be taken forward to the density functional theory (DFT) optimisation step.

5. Crystal structure optimisation

For the structural relaxations, we employ DFT with a projector-augmented plane wave basis[4] and the PBEsol exchange-correlation functional[5] as implemented in the Vienna Ab-initio Simulation Package (VASP)[6,7]. A Monkhorst-Pack k-point grid was generated for each calculation with k-point spacing of 0.242 $Å^{-1}$. The kinetic energy cutoff is set at 500 eV and the force on each atom is converged to within 0.01 $eVÅ^{-1}$. For each of the four element combinations, the lowest total energy structure of those for which a local minimum could be found was taken forward to the bandgap calculation step.

6. Electronic structure calculations

Semi-local exchange-correlation treatments such as the PBEsol functional provide an accurate description of crystal structures but tend to underestimate the electronic bandgaps of semiconductors. To overcome this issue, computations of bandgaps are performed by

using the hybrid non-local functional HSE06,[8] which includes 25% screened Hartree-Fock exact exchange. The calculated bandgaps of the four final materials are presented in Table S2.

REFERENCES

[1]O. Madelung, *Semiconductors: Data Handbook* (Springer-Verlag, Berlin, Heidelberg, 2004).

[2]B. D. Pelatt, R. Ravichandran, J. F. Wager, and D. A. Keszler, J. Am. Chem. Soc. **133**, 16852 (2011).

[3]G. Hautier, C. Fischer, V. Ehrlacher, A. Jain, and G. Ceder, Inorg. Chem. **50**, 656 (2011).

[4]G. Kresse and D. Joubert, Phys. Rev. B **59**, 1758 (1999).

[5]J. P. Perdew, A. Ruzsinszky, G. I. Csonka, O. A. Vydrov, G. E. Scuseria, L. A. Constantin, X. Zhou, and K. Burke, Phys. Rev. Lett. **100**, 136406 (2008).

[6]G. Kresse and J. Furthmüller, Comput. Mater. Sci. **6**, 15 (1996).

[7]G. Kresse and J. Furthmüller, Phys. Rev. B **54**, 11169 (1996).

[8]A. V. Krukau, O. A. Vydrov, A. F. Izmaylov, and G. E. Scuseria, J. Chem. Phys. **125**, 224106 (2006).
