# Supplementary material for: Computational Screening of All Stoichiometric Inorganic Materials
Source: Chem. 2016 Oct 13;1(4):617–27. doi: 10.1016/j.chempr.2016.09.010 (PMC5074417; doi:10.1016/j.chempr.2016.09.010)
Supplement: Document S2. Article plus Supplemental Information [file mmc2.pdf]

Chem

CellPress

Article

Computational Screening of All Stoichiometric Inorganic Materials

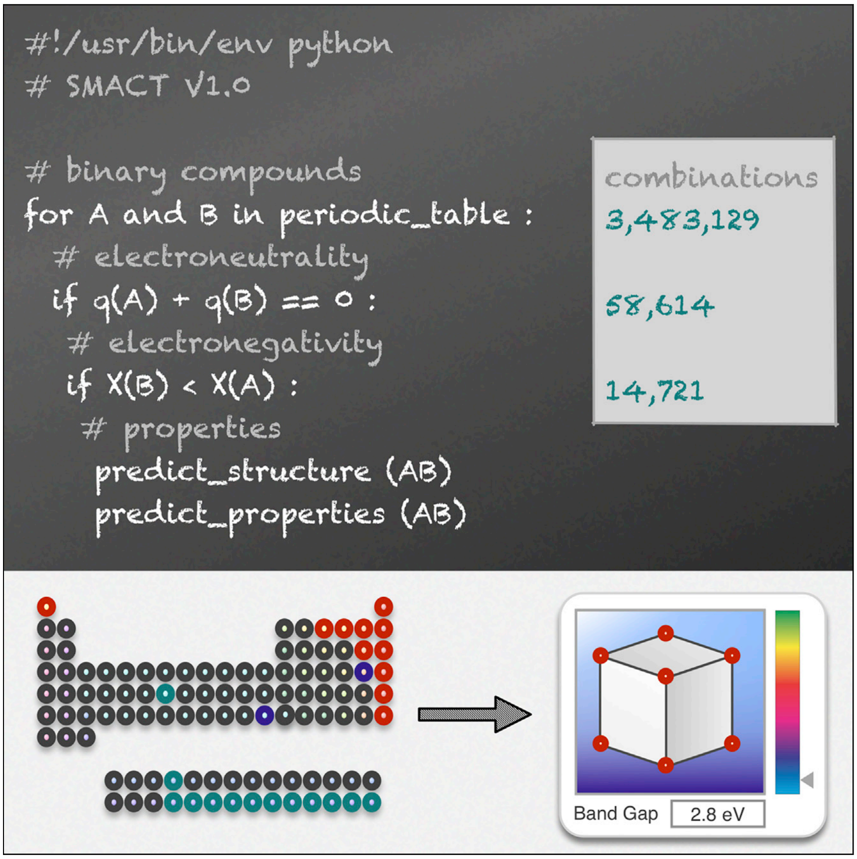

The compositional space for inorganic materials remains vastly unexplored. Walsh and colleagues have designed procedures that use well-established chemical knowledge to quantify the number of possible multi-component compounds. They show how chemical filters can be applied to quickly and effectively narrow down the number of results and focus on those with target functionality.

Daniel W. Davies, Keith T. Butler, Adam J. Jackson, Andrew Morris, Jarvist M. Frost, Jonathan M. Skelton, Aron Walsh

a.walsh@imperial.ac.uk

HIGHLIGHTS

Compositional space for two- to four-component inorganic materials is quantified

Novel compounds for water splitting are identified by a low-cost screening procedure

A large number of possible perovskite-structured materials are discovered

SMACT, an open-source material-screening Python package, is reported

7 AFFORDABLE AND CLEAN ENERGY

Davies et al., Chem 1, 617–627
October 13, 2016 © 2016 The Author(s).
Published by Elsevier Inc.
http://dx.doi.org/10.1016/j.chempr.2016.09.010

CrossMark

Chem

CellPress

Article

Computational Screening of All Stoichiometric Inorganic Materials

Daniel W. Davies,[1,4] Keith T. Butler,[1,4] Adam J. Jackson,[1,4] Andrew Morris,[1] Jarvist M. Frost,[1] Jonathan M. Skelton,[1] and Aron Walsh[1,2,3,5,*]

SUMMARY

Forming a four-component compound from the first 103 elements of the periodic table results in more than 10^{12} combinations. Such a materials space is intractable to high-throughput experiment or first-principle computation. We introduce a framework to address this problem and quantify how many materials can exist. We apply principles of valency and electronegativity to filter chemically implausible compositions, which reduces the inorganic quaternary space to 10^{10} combinations. We demonstrate that estimates of band gaps and absolute electron energies can be made simply on the basis of the chemical composition and apply this to the search for new semiconducting materials to support the photoelectrochemical splitting of water. We show the applicability to predicting crystal structure by analogy with known compounds, including exploration of the phase space for ternary combinations that form a perovskite lattice. Computer screening reproduces known perovskite materials and predicts the feasibility of thousands more. Given the simplicity of the approach, large-scale searches can be performed on a single workstation.

The Bigger Picture

The discovery of functional materials is critical for technological advancements that will play a role in addressing global challenges, ranging from catalysis for sanitation, semiconductors for harvesting solar energy, and biomimetic materials for health. There is a concerted global effort to reduce the time it takes to realize new materials via databases, high-throughput screening, informatics, and mapping out the "materials genome."

Here, we show how the compositional space for stoichiometric, inorganic materials can be quantified by simple rules and how the vast space can be explored quickly and cheaply with the use of key chemical concepts and element properties in the search for candidate materials with target properties. We exemplify the application of this approach by identifying a chalcohalide material with potential for water-splitting applications and carrying out a comprehensive search for new compositions that could adopt the widely studied perovskite crystal structure.

INTRODUCTION

Currently, over 184,000 entries in the Inorganic Crystal Structure Database (ICSD) involve 9,141 structure types;[1] 66,814 of these materials have also been subject to quantum mechanical calculations, and information on their basic electronic structures and thermodynamics is included in the Materials Project[2] (powered by the PYMATGEN infrastructure[3]).

The configurational phase space for new materials is immense, but blind exploration of the periodic table is a daunting task. Fortunately, over a century of research in the physical sciences has provided us with myriad rules for assessing the feasibility of a given stoichiometry and the likelihood of particular crystal arrangements. Examples of chemical phenomenology include the radius ratio rules[4] and Pettifor maps[5] for structure prediction, as well as electronegativity and chemical hardness for predicting reactivity.[6] Pauling's rules[7] provide predictive power for ionic or heteropolar crystals. A wealth of knowledge exists for understanding the physical properties of tetrahedral semiconductors.[8] Recent examples of searches for new materials that draw from existing chemical knowledge include 18-electron ABX compounds,[9] hyperferroelectric superlattices,[10] and organic-inorganic perovskites.[11,12]

The reliability and predictive power of atomistic material simulations is increasing.[13,14] Many approximations are being removed as high-performance supercomputers reach petaflop scale. This includes more accurate quantum

CrossMark

This is an open access article under the CC BY license (http://creativecommons.org/licenses/by/4.0/).

mechanical treatment of electron-electron interactions in the solid state,[15] as well as more realistic models of chemical disorder.[16] However, because of the computational cost, high-throughput screening with first-principle techniques is usually limited to hundreds or thousands of materials—a small fraction of the overall phase space.

We report a procedure for navigating the materials landscape with low computational effort, and it can be achieved with simple chemical descriptors. We first explore the magnitude of the task at hand by enumerating combinations of elements and ions for binary, ternary, and quaternary compositions. We demonstrate that chemical constraints can narrow the search space drastically. Examples of how deeper insights can be gained are illustrated for electronic (photoelectrodes for water splitting) and structured (perovskite-type) materials. The procedure can be used to comfortably explore the vast compositional space or as the first step in a multi-stage high-throughput screening process. Instead of being a roadblock to achieving new functionality, the combinatorial explosion for multi-component compounds provides fertile ground for the discovery of innovative materials.

RESULTS

Elemental Combinations

To begin, one can map chemical space by enumerating the ways in which the constituent elements of the periodic table can combine. If we restrict ourselves to the first 103 elements (to the end of the actinide series), the combinations (i.e., C_n^{103}) for two, three, and four components are 5,253, 176,851, and 4,421,275, respectively. For five components, the combinations exceed 87 million.

Physically, the situation is more complex. Elements can combine in different ratios, leading to variation in material stoichiometry, e.g., the binary combinations AB, AB_2, A_2B_3, and A_3B_4. Given elements can also adopt multiple oxidation states, each with a unique chemical behavior, e.g., Sn(II)O, Sn(IV)O_2, and Sn(II)Sn(IV)O_3. For our enumeration of feasible compounds, we next consider the accessible oxidation states of each element in stoichiometry up to quaternary $A_wB_xC_yD_z$, where the integers w, x, y, and $z \leq 8$. This definition includes, for example, common ternary pyrochlore oxides ($A_2B_2O_7$) and quaternary double perovskites (A_2BCO_6). Using the most common oxidation states extends the first 103 elements of the periodic table to 403 unique ions.

The number of combinations is now drastically increased, as shown in Table 1, such that four-component candidate materials exceed 10^{12}. In order to reduce this composition space, we can introduce selection rules (filters) from chemical theory.

We note that the estimations discussed here represent a lower limit on the number of accessible materials. We consider regular inorganic compounds and exclude, for example, non-stoichiometry, organic systems, hybrid organic-inorganic materials, electrides, and intermetallics, for which additional considerations are required for predicting viability.[17–19]

Chemical Filters

Rule 1: Charge Neutrality

Ions tend to combine into charge-neutral aggregates. The same thinking applies to both ionic solids and more covalently bonded semiconductors. Any periodic solid must be charge neutral; otherwise, it would have an infinite electrostatic potential. Balancing oxidation states and fulfilling the valence octet rule are equivalent,

[1]Department of Chemistry, Centre for Sustainable Chemical Technologies, University of Bath, Claverton Down, Bath BA2 7AY, UK

[2]Department of Materials Science and Engineering, Global E[3] Institute, Yonsei University, Seoul 120-749, Korea

[3]Department of Materials, Imperial College London, Exhibition Road, London SW7 2AZ, UK

[4]Co-first author

[5]Lead Contact

*Correspondence: a.walsh@imperial.ac.uk

http://dx.doi.org/10.1016/j.chempr.2016.09.010

Table 1. Estimates for the Number of Possible Inorganic Materials Allowing for Variable Oxidation States and Stoichiometry with the Constraints of Charge Neutrality and Electronegativity Balance

Type	Constraint[a]	Number
A_wB_x	–	3,483,129
A_wB_x	q	58,614
A_wB_x	$q + \chi$	14,721
$A_wB_xC_y$	–	4,753,229,039
$A_wB_xC_y$	q	174,081,685
$A_wB_xC_y$	$q + \chi$	32,157,899
$A_wB_xC_yD_z$	–	4,139,315,402,300
$A_wB_xC_yD_z$	q	267,381,955,246
$A_wB_xC_yD_z$	$q + \chi$	32,381,953,858

[a] q, charge neutralitiy; X, electronegativity balance.

e.g., III–V semiconductors, such as GaAs, can be represented as $Ga^{3+}As^{3-}$. Our implementation is inspired by the work of Pamplin[20] and Goodman[21] on the subject of multi-component semiconductors.

A charge-neutrality constraint significantly reduces the total number of candidate materials. The rule states that the formal charges (q) of the components sum to 0, i.e.,

$$wq^A + xq^B + yq^C + zq^D = 0. \qquad \text{(Equation 1)}$$

Charge neutrality contracts the compositional space by at least an order of magnitude for binaries, ternaries, and quaternaries (Table 1).

Rule 2: Electronegativity Balance

Further to assuming that all charge-neutral combinations of oxidation states are accessible, we can implement a second constraint based on the electronegativity of the component elements. The empirical electronegativity (χ) scale represents the "attraction" of a particular atom for electrons. For a stable compound, the relation $\chi^{cation} < \chi^{anion}$ should be obeyed, i.e., the most electronegative element carries the most negative charge. Here, we employ the Pauling electronegativity scale, which reduces the allowed compositions by a factor of between 4 and 10 for the different numbers of components (Table 1).

It is also instructive to consider existing materials databases (the ICSD and Materials Project). For binary compounds, we find fewer combinations from our estimates as implemented in SMACT (Semiconducting Materials from Analogy and Chemical Theory) than from the ICSD (Figure 1), which can largely be attributed to our exclusion of intermetallics and polymorphs. In the Materials Project, multiple entries for a single crystal structure and chemical composition are removed, and the number of compositions are in close agreement. For ternaries and quaternaries, the compositions passing both charge and electronegativity tests continue to rise exponentially, whereas the number in existing databases remains relatively constant. The increased complexity of ternary and quaternary systems means that their synthesis, characterization, and reporting are more challenging than for binary systems. Nevertheless, the large differences between the numbers of potential and reported materials suggest that wide areas of unexplored compositional space may contain stable and useful materials.

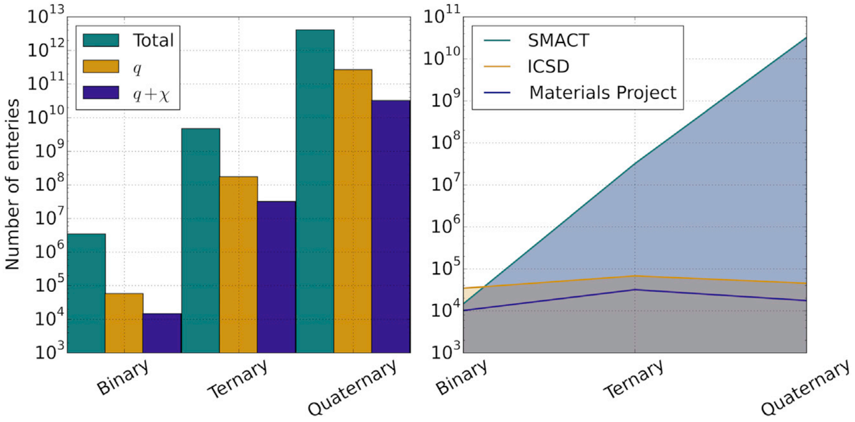

Figure 1. Counting the Number of Possible Multi-component Materials

(Left) Narrowing of compositional space for inorganic materials by imposing chemical constraints of charge (q) and electronegativity (χ).

(Right) Comparison of the accessible materials predicted by SMACT and those reported in the ICSD[1] and the Materials Project.[2]

The numbers reported in this section are vast, and using modern electronic-structure techniques to perform quantitative screening for application is unimaginable. Exploration of the hitherto neglected compositional space will require further guidelines. In the following sections, we demonstrate how additional descriptors can be applied for identifing materials for specific applications.

Compositional Descriptors

Several useful properties can be estimated from knowledge of the chemical composition alone, and here we explore the application of some of these approaches.

Descriptor 1: Electronic Chemical Potential

The concept of atomic electronegativity has been successfully extended to solids, where the geometric mean becomes the single-value descriptor, i.e.,

$$\chi^{\mathrm{solid}} = \sqrt[w+x+y+z]{\chi_A^w \chi_B^x \chi_C^y \chi_D^z}\,. \qquad \text{(Equation 2)}$$

This descriptor represents a mid-gap energy between the filled (valence band) and empty (conduction band) electronic states. This corresponds to the electronic chemical potential (Fermi level) at 0 K.[22] Butler and Ginley[23] found a linear correlation between the solid electronegativity and the electrochemical flat-band potentials for a range of semiconductors. This was subsequently extended to a wider dataset including metal oxides, chalcogenides, and halides.[24] The method provides a rapid procedure for the estimation of absolute electron energies for multi-component materials. It is now commonly used in the computational screening of new materials for electrochemical applications.[25–28]

Descriptor 2: Electronic Structure

Many tight-binding model Hamiltonians exist for semiconductors and dielectrics.[8] One recent approach is based on the atomic solid-state energy (SSE) scale,[29] which provides information on valence and conduction bands on the basis of the frontier orbitals of the constituent ions. Whereas the Mulliken definition of electronegativity is an average of the ionization potential (IP) and electron affinity (EA) of an atom, the SSE reflects the IP of an anion (filled electronic states) and EA of a cation (empty electronic states). The energies of 40 elements were fitted from a test set of 69

closed-shell binary inorganic semiconductors,[29] which has recently been extended to 94 elements.[30] According to the tabulated SSE scale, the band gap (E_g) can be estimated as

$$E_g^{SSE} = SSE^{cation} - SSE^{anion}. \quad \text{(Equation 3)}$$

For multi-component systems, the limiting values (cation with highest EA and anion with lowest IP) are used. The SSE has a root-mean-square deviation of 0.66 eV against the measured band gaps of 35 ternary semiconductors (see Table S1). This simple method allows for rapid screening of band gaps and absolute band-edge alignment.

Both methods (Equations 2 and 3) have been implemented for arbitrary compositions on the basis of tabulated atomic data in the SMACT package. Because no crystal-structure information is included at this level, the results are qualitative, and the models do not distinguish, for example, between polymorphs.

Electronic Structure: Photoelectrodes

We now use the compositional space and chemical descriptors defined above to search for potential materials for solar fuel generation via photoelectrochemical water splitting.

The properties that are required for viable photoelectrodes include (1) a band gap in the visible range of the electromagnetic spectrum to absorb a significant fraction of sunlight and (2) upper valence and lower conduction bands bridging the water oxidation and reduction potentials, enabling the redox reaction. We set an optimal band-gap range between 1.5 and 2.5 eV. Although the free energy for water dissociation is 1.2 eV, the combination of loss mechanisms found in practical devices could require a band gap as large as 2.2 eV.[31,32]

Metal oxides combine many attractive properties for water splitting (e.g., stability and cost), but they usually have band gaps too large to absorb a significant fraction of sunlight. The formation of multi-anion compounds offers a route to modifying the electronic structure. We consider ternary metal chalcohalides (i.e., $A_xB_yC_z$), with B = [O,S,Se,Te] and C = [F,Cl,Br,I]. We restrict the A cations to those with an SSE higher than the water reduction potential (approximately −4.5 V in relation to the vacuum at pH = 0). The conditions of charge neutrality and electronegativity are used for performing an initial screening that yields 52,094 combinations. With the additional band-gap criterion, the combinations are reduced to 7,676, and the pool of cations is reduced from 25 to 7 with A = [B,Ti,V,Zn,Ga,Cd,Sn]. We further rule out any boron-containing combinations at this stage, because these are known to form discrete molecular units (e.g., BClSe).

Finally, we screen compositions according to the environmental sustainability of the elements. We use the Herfindahl-Hirschman Index (HHI_R), which has been developed in the context of thermoelectric applications, for elemental reserves.[33] This index includes factors such as geopolitical influence over materials supply and price. The HHI_R for a given composition can be obtained as the weighted average over the constituent elements. At this stage, because stoichiometry is variable, we consider the mean HHI_R for each $A_1B_1C_1$ combination.

The band-edge positions of the 20 candidates with the smallest HHI_R values are presented in Figure 2. The HHI_R has the effect of eliminating all combinations containing

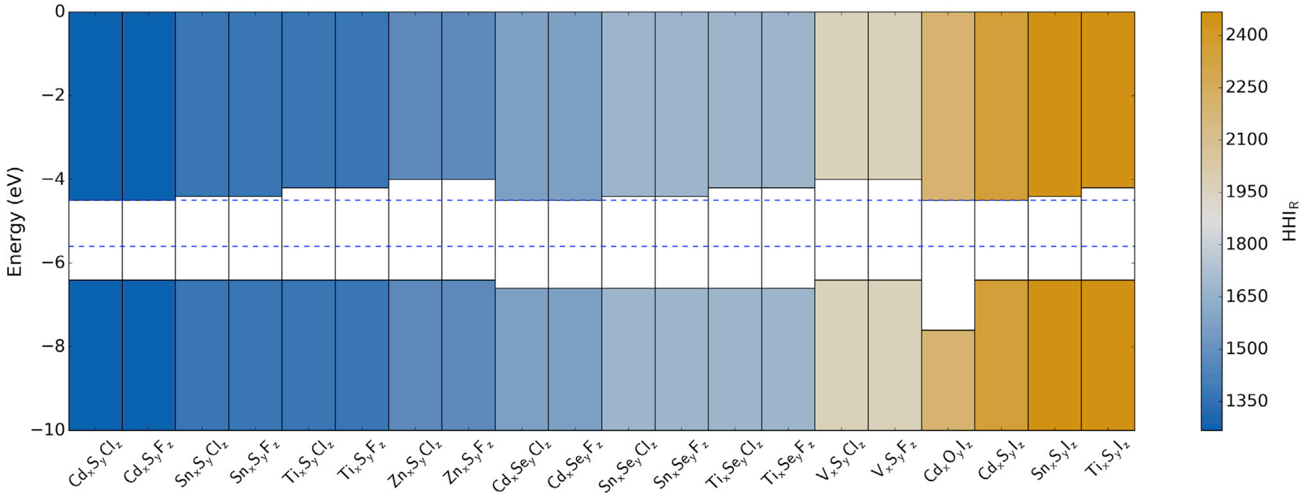

Figure 2. Calculated Band-Edge Positions of 20 Promising Element Combinations for Water-Splitting Applications

Band-edge positions were calculated in relation to the vacuum level on the basis of the solid-state energies of the constituent elements. Blue dashed lines indicate the water reduction (above) and oxidation (below) potentials with respect to vacuum.

Ga, Te, and Br (although relatively abundant, most of the world's Br is produced from the Dead Sea, making it geopolitically sensitive, as reflected in a high HHI_R). There are no entries in the ICSD for most of the candidates that we identified; however, reports can be found for $Cd_2O_6I_2$, Sn_2SI_2, and $Zn_6S_5Cl_2$.[34–36] Both $Cd_2O_6I_2$ and Sn_2SI_2 feature in the Materials Project and have band gaps of 3.3 and 1.6 eV, respectively, calculated within density functional theory (DFT). These compare with the SSE band gaps of 2.5 and 2.0 eV. The third compound, $Zn_6S_5Cl_2$, is reported to have an optical gap of 2.7 eV[36], which compares with the SSE band gap of 2.4 eV.

Only one oxygen-containing compound survived the band-gap screening criterion; the values for metal oxyhalides are generally too large. For O_yI_z, the iodide forms the upper valence band (low binding energy of I 5p), whereas it is the oxide (O 2p) for other halides. However, the sensitivity of the oxide ion to its crystal environment is well documented,[27,37] and consequently its SSE carries the greatest uncertainty.[29] This is one aspect where knowledge of the local structure (electrostatic potential) could significantly improve the accuracy of the results.

We must connect composition to crystal structure in order to make more accurate property predictions. Global optimization of crystal structures from first principles is a formidable task, although great progress is being made in this area.[38] We instead adopt an approach based on analogy with known structures through chemical substitutions, as developed by Hautier et al.[39] It uses data-mined probability functions, as implemented in the Materials Project.

To demonstrate the translation from composition to material, we performed crystal-structure mining for the four combinations with the lowest HHI_R. The 88 predicted structures were then subjected to a full DFT lattice optimization procedure and ranked by total internal energy. Finally, accurate band gaps were predicted for the lowest-energy structures by hybrid DFT (HSE06 electron exchange and correlation[40,41]). The compound $Sn_5S_4Cl_2$ has an indirect band gap of 1.6 eV and a direct gap of 1.8 eV, which lies within the target range. The band gaps of the other three

lowest-energy compounds were calculated to be between 3.0 and 3.4 eV. Full details of the workflow (Figure S1) and band gaps (Table S2) can be found in the Supplemental Information.

The newly identified compound, $Sn(II)_5S_4Cl_2$, adopts a structure formed from two distinct Sn centered polyhedra: (1) a distorted octahedron with equatorial S and apical Cl ions and (2) a distorted tetrahedron with 4 S ions and a stereochemically active Sn lone pair (Figure S2). The polyhedra form interlocking chains in three dimensions. The electronic density of states reveals an upper valence band composed of hybridized Sn *s* – Cl *p* orbitals; such Sn *s*-based valence bands are considered promising indicators for hole mobility.[42] The lower conduction band is composed mainly of overlapping Sn *p* orbitals. The chemical structure and bonding characteristics suggest that this material should have favorable carrier transport, crucial for optoelectronic applications.

Crystal Structure: Perovskites

One of the most successful approaches to discovering new materials is structural analogy. The concept is to take a crystal structure with a known chemistry and to replace elements within the structure to tune the physical properties. In the simplest case, this involves direct isovalent substitution, e.g., Zn(II)S → Cd(II)S. Structural analogy can be extended to aliovalent cross-substitution (also termed cation mutation), e.g., Zn(II)S → $Cu(I)Ga(III)S_2$. A systematic methodology was outlined more than 40 years ago in a paper by Pamplin[20] for enumerating charge-neutral tetrahedral semiconductors.

The challenge of going beyond tetrahedral semiconductors is predicting crystal structure. The radius of ions within a lattice has a long history as a geometric descriptor of structural stability. A key example is the application of radius ratio rules by Goldschmidt[43] to predict the propensity of a ternary ABC_3 combination to form the perovskite structure:

$$t = \frac{r^A + r^C}{\sqrt{2}(r^B + r^C)}, \qquad \text{(Equation 4)}$$

where t is the tolerance factor and r is the ionic radius. Values of $t > 1$ imply a relatively large A site favoring a hexagonal structure, $0.9 < t < 1$ predicts a cubic structure, and $0.7 < t < 0.9$ means that the A site is small, preferring an orthorhombic structure. For $t < 0.7$, other (non-perovskite) structures are predicted. These rules have recently been extended to describe structure-property relationships in hybrid organic-inorganic perovskites.[11,12]

In this section, we apply our screening procedure to include knowledge of the crystal structure and estimate the size of the perovskite materials space. We start by enumerating the elemental combinations. We then reduce the set by requiring an octahedral coordination environment for the B site, as contained in the Shannon dataset,[44] and require a combination of oxidation states that are charge neutral. This list is then assessed in terms of t, as defined by the Shannon ionic radii.[44]

We consider single-anion compositions based on C = [O,S,Se,F,Cl,Br,I]. The charge-neutrality and octahedral B-site constraints reduce the 176,851 elemental combinations to 41,725. The tolerance-factor constraint, $0.7 < t < 1.0$, further reduces this to 26,567. For potential applications in the energy sector, we can consider candidates with HHI_R smaller than that of CdTe (a commercial thin-film photovoltaic material), resulting in a final population of 13,415.

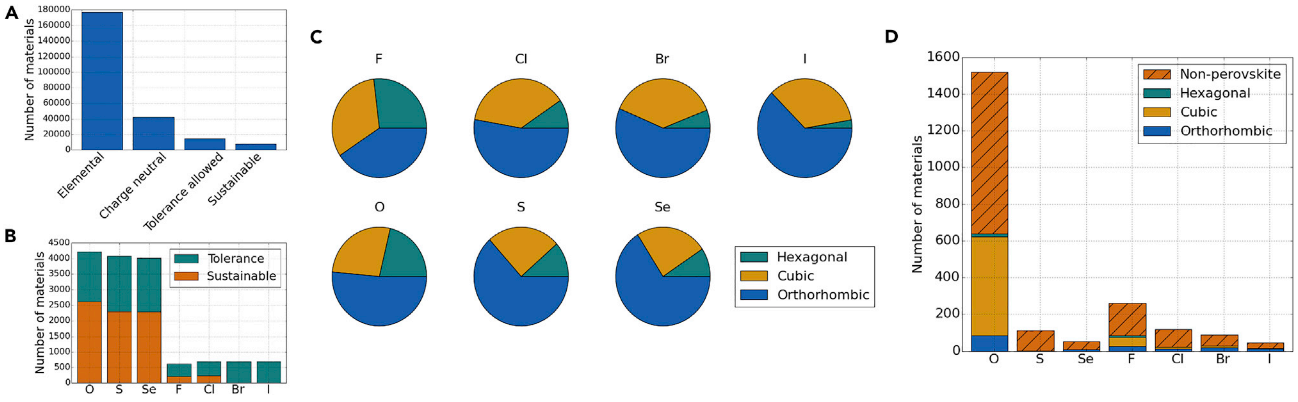

Figure 3. Counting Experiments with Perovskites

(A) Combinations found at each stage of the screening procedure.

(B) Perovskite compounds with an HHI_R lower than CdTe (3,296) for each anion.

(C) The distribution of hexagonal, cubic, and orthorhombic perovskite structures predicted on the basis of the Goldschmidt tolerance factor and Shannon radii of the ions.

(D) ABC_3 combinations found in the Materials Project database. They are sorted into structure type according to space group (here, orthorhombic and lower-symmetry perovskites are grouped together).

For each anion, an orthorhombic perovskite structure is the most common prediction, and hexagonal is the most rare (Figure 3). The fraction of cubic perovskite structures remains roughly constant within the respective halide and oxide or chalcogenide series; however, it is more dominant for the halides. The presence of Br or I makes a material less sustainable (higher HHI_R); otherwise, there is little to differentiate the anions.

Far more oxide and chalcogenide perovskites are predicted than halides. The higher anion charge allows for three distinct cation combinations (I-V, II-IV, and III-III), whereas halides have only I-II. In addition, a greater radius compatibility is found for the group VI anions. We find that the number of plausible perovskite structures increases with the anion radius; however, the lower crustal abundance for heavier elements reduces the number that meet the sustainability criterion.

A search of the Materials Project over the same anion space reveals 920 materials, a small fraction of those predicted from SMACT (26,567). The search includes all standard perovskite space groups.[45] For oxide perovskites, 8.26% of those identified from SMACT are found in the Materials Project; for sulfides, this falls to 0.45% and to 0.12% for selenides. To some extent, the greater number of oxide perovskites discovered reflects the greater research activity in this field; however, synthesis of chalogenide perovskites has been reported,[46–48] and there is interest in these materials for technological applications.[49,50] Of the ABC_3 materials reported in the Materials Project, 48% of oxides, 35% of sulfides, and 20% of selenides are in perovskite space groups.

Why are there so few chalcogenide perovskites? The tolerance-factor arguments that work well for metal oxides may not hold for chalcogenide perovskites. Oxygen forms more ionic compounds because of its higher electronegativity and lower polarizability than those of S, Se, and Te. When covalent bonding becomes prevalent, it is known to result in deviations from tolerance-factor behavior. An example is the case of $NaSbO_3$, for which $t = 0.92$ is commensurate with the formation of cubic perovskite but which forms the non-perovskite ilmenite structure. Goodenough and Kafalas[51] explained this deviation as a result of strong σ bonding between Sb and O.

Table 2. Data Sources for SMACT

Data Type	Source
Abundance	estimated crustal abundance of elements from the CRC Handbook of Physics and Chemistry[53]
Atomic mass	NIST Standard Reference Database 144;[54] where the relative abundance of isotopes was unknown or a range of values was provided, a simple mean was taken
Covalent radius	scientific paper[55]
Electron affinity	scientific paper;[56] no default value was used for elements that lack data on electron affinity
Eigenvalues	highest occupied p-state and s-state eigenvalues were tabulated by Harrison[57] from the approximate Hartree-Fock calculations of Herman and Skillman[58]
HHI	elemental Herfindahl-Hirschman Index calculated from geological and geopolitical data[33]
Ionization potential	NIST Atomic Spectra Database[59]
Pauling electronegativity	updated values of electronegativity on Pauling's scale were compiled in the CRC Handbook;[53] for elements 95 (Am) and above, Pauling's recommended value of 1.3 was used;[60] the value for krypton (3.0) was derived from the bond energy of KrF_2 and reported in a scientific paper[61]
SSE	"solid-state energy" model of semiconductors and dielectrics[29,30]
SSE (Pauling)	extended estimates of solid-state energy from the correlation between known values and Pauling electronegativity[30]

Where possible, values recommended by the National Institute of Standards and Technology (NIST) were used.

This procedure demonstrates the power of searching through materials on the basis of structural analogy. Only a small fraction of possible perovskite materials have been synthesized. Although some may not represent thermodynamic ground states, they could be accessible through kinetic control of crystal growth or templated on a substrate. Many interesting chalcogenide perovskites are waiting to be discovered. The final pool of 13,415 feasible compositions is within the grasp of explicit computation by quantum mechanical methods, albeit as part of an ambitious project. Indeed, high-throughput screening of 5,400 multi-anion cubic perovskite structures via DFT has been reported[25,52] and revealed 32 promising new materials for water-splitting applications.

Conclusions

We have demonstrated the utility of chemical theory in quantifying the magnitude of the compositional space for multi-component inorganic materials. Even after the application of chemical filters, the space for four-component materials exceeds 10^{10} combinations. We further estimate that the five-component space exceeds 10^{13} combinations. There are many applications in which materials with even higher-order compositions have been developed, e.g., in high-temperature superconductors, where six to seven component materials are common. The number of potential materials is not infinite, but it is immense. The scale of the combinatorial explosion emphasizes the need for effective material-design procedures that employ existing chemical and physical knowledge in a targeted manner. Stochastic sampling of this chemical space is unlikely to be effective in yielding materials with specific functionality. We have presented a procedure that uses simple descriptors to support materials exploration, discovery, and design.

EXPERIMENTAL PROCEDURES

Code Implementation

SMACT, the Python toolkit developed in this work, is available online at https://github.com/WMD-group/SMACT. It is free software made available under the Gnu Public License (GPL) version 3.

All element counts and plots presented in this paper were created with custom codes based on SMACT and written in the Python 2.7 programming language. Elemental data are collated from multiple sources (see Table 2) and made

algorithmically accessible in a unified object-orientated interface. Example routines that check element and oxidation-state combinations against the conditions of charge neutrality and electronegativity are provided.

Scripts that generate the results and plots reported in this paper are available with the SMACT codes. A number of tutorials working through the combinatorial explosion are provided at https://github.com/WMD-group/SMACT_practical.

The codes, collectively named Semiconducting Materials by Analogy and Chemical Theory, are inspired by the pen-and-paper procedure reported by Pamplin in 1964.[20]

SUPPLEMENTAL INFORMATION

Supplemental Information includes Supplemental Experimental Procedures, two figures, and two tables and can be found with this article online at http://dx.doi.org/10.1016/j.chempr.2016.09.010.

AUTHOR CONTRIBUTIONS

All authors contributed to the development of the SMACT package; the primary coding was performed by K.T.B., A.J.J., and D.W.D. A.W., D.W.D. and K.T.B. wrote the first draft of the manuscript with input, discussion, and analysis from all co-authors.

ACKNOWLEDGMENTS

We acknowledge the contributions of T. Gauntlett and J. Evans to the addition of Shannon radii and sustainability data in SMACT. This research was supported by the Royal Society, European Research Council (grant no. 277757), and the EPSRC (EP/J017361/1, EP/K004956/1, EP/K016288/1, EP/L017792/1, EP/M009580/1, and EP/G03768X/1).

Received: May 5, 2016
Revised: June 28, 2016
Accepted: September 21, 2016
Published: October 13, 2016

REFERENCES AND NOTES

1. Royal Society of Chemistry. CDS: National Chemical Database Service. http://icsd.cds.rsc.org.

2. The Materials Project. https://www.materialsproject.org.

3. Ong, S.P., Richards, W.D., Jain, A., Hautier, G., Kocher, M., Cholia, S., Gunter, D., Chevrier, V.L., Persson, K.A., and Ceder, G. (2013). Python Materials Genomics (pymatgen): a robust, open-source python library for materials analysis. Comput. Mater. Sci. *68*, 314–319.

4. Goldschmidt, V.M. (1926). Die Gesetze der Krystallochemie. Naturwissenschaften *14*, 477–485.

5. Pettifor, D.G. (1986). The structures of binary compounds. I. Phenomenological structure maps. J. Phys. C Solid State Phys. *19*, 285–313.

6. Pearson, R.G. (1988). Absolute electronegativity and hardness: application to inorganic chemistry. Inorg. Chem. *27*, 734–740.

7. Pauling, L. (1929). The principles determining the structure of complex ionic crystals. J. Am. Chem. Soc. *51*, 1010–1026.

8. Phillips, J. (1973). Bonds and Bands in Semiconductors (Academic Press).

9. Gautier, R., Zhang, X., Hu, L., Yu, L., Lin, Y., Sunde, T.O., Chon, D., Poeppelmeier, K.R., and Zunger, A. (2015). Prediction and accelerated laboratory discovery of previously unknown 18-electron ABX compounds. Nat. Chem. *7*, 308–316.

10. Garrity, K.F., Rabe, K.M., and Vanderbilt, D. (2014). Hyperferroelectrics: proper ferroelectrics with persistent polarization. Phys. Rev. Lett. *112*, 127601.

11. Kieslich, G., Sun, S., and Cheetham, T. (2015). An extended tolerance factor approach for organic-inorganic perovskites. Chem. Sci. *6*, 3430–3433.

12. Travis, W., Glover, E.N.K., Bronstein, H., Scanlon, D.O., and Palgrave, R. (2016). On the application of the tolerance factor to inorganic and hybrid halide perovskites: a revised system. Chem. Sci. *7*, 4548–4556.

13. Lejaeghere, K., Bihlmayer, G., Björkman, T., Blaha, P., Blügel, S., Blum, V., Caliste, D., Castelli, I.E., Clark, S.J., Dal Corso, A., et al. (2016). Reproducibility in density-functional theory calculations of solids. Science *351*, 1–11.

14. Butler, K.T., Frost, J.M., Skelton, J.M., Svane, K.L., and Walsh, A. (2016). Computational materials design of crystalline solids. Chem. Soc. Rev. http://dx.doi.org/10.1039/C5CS00841G.

15. Booth, G.H., Grüneis, A., Kresse, G., and Alavi, A. (2013). Towards an exact description of electronic wavefunctions in real solids. Nature *493*, 365–370.

16. Liu, J., Fernández-Serra, M.V., and Allen, P.B. (2016). Special quasiordered structures: role of short-range order in the semiconductor alloy $(GaN)_{1-x}(ZnO)_x$. Phys. Rev. B *93*, 054207.

17. Kieslich, G., Kumagai, S., Butler, K.T., Okamura, T., Hendon, C.H., Sun, S., Yamashita, M., Walsh, A., and Cheetham, A.K. (2015). Role of entropic effects in controlling the polymorphism in formate ABX_3 metal-organic frameworks. Chem. Commun. *51*, 15538–15541.

18. Jiang, C., and Uberuaga, B.P. (2016). Efficient *ab initio* modeling of random multicomponent alloys. Phys. Rev. Lett. *116*, 105501.

19. Caetano, C., Butler, K.T., and Walsh, A. (2016). Analysis of electrostatic stability and ordering in quaternary perovskite solid solutions. Phys. Rev. B *93*, 144205.

20. Pamplin, B. (1964). A systematic method of deriving new semiconducting compounds by structural analogy. J. Phys. Chem. Solids *25*, 675–684.

21. Goodman, C.H.L. (1958). The prediction of semiconducting properties in inorganic compounds. J. Phys. Chem. Solids *6*, 305–314.

22. Nethercot, A.H. (1974). Prediction of Fermi energies and photoelectric thresholds based on electronegativity concepts. Phys. Rev. Lett. *33*, 1088.

23. Butler, M.A., and Ginley, D.S. (1978). Prediction of flatband potentials at semiconductor-electrolyte interfaces from atomic electronegativities. J. Electrochem. Soc. *125*, 228.

24. Yong, X., and Schoonen, M.A.A. (2000). The absolute energy positions of conduction and valence bands of selected semiconducting minerals. Am. Mineral. *85*, 543–556.

25. Castelli, I.E., Landis, D., Thygesen, K.S., Dahl, S., Chorkendorff, I., Jaramillo, T.F., and Jacobsen, K.W. (2012). New cubic perovskites for one- and two-photon water splitting using the computational materials repository. Energy Environ. Sci. *5*, 9034–9043.

26. Burton, L.A., and Walsh, A. (2012). A photoactive titanate with a stereochemically active Sn lone pair: electronic and crystal structure of Sn_2TiO_4 from computational chemistry. J. Solid State Chem. *196*, 157–160.

27. Walsh, A., and Butler, K.T. (2014). Prediction of electron energies in metal oxides. Acc. Chem. Res. *47*, 364–372.

28. Stevanović, V., Lany, S., Ginley, D.S., Tumas, W., and Zunger, A. (2014). Assessing capability of semiconductors to split water using ionization potentials and electron affinities only. Phys. Chem. Chem. Phys. *16*, 3706–3714.

29. Pelatt, B.D., Ravichandran, R., Wager, J.F., and Keszler, D.A. (2011). Atomic solid state energy scale. J. Am. Chem. Soc. *133*, 16852–16860.

30. Pelatt, B.D., Kokenyesi, R.S., Ravichandran, R., Pereira, C.B., Wager, J.F., and Keszler, D.A. (2015). Atomic solid state energy scale: universality and periodic trends in oxidation state. J. Solid State Chem. *231*, 138–144.

31. Bak, T., Nowotny, J., Rekas, M., and Sorrell, C.C. (2002). Photo-electrochemical hydrogen generation from water using solar energy. Materials-related aspects. Int. J. Hydrogen Energy *27*, 991–1022.

32. Pinaud, B.A., Benck, J.D., Seitz, L.C., Forman, A.J., Chen, Z., Deutsch, T.G., James, B.D., Baum, K.N., Baum, G.N., Ardo, S., et al. (2013). Technical and economic feasibility of centralized facilities for solar hydrogen production via photocatalysis and photoelectrochemistry. Energy Environ. Sci. *6*, 1983–2002.

33. Gaultois, M.W., Sparks, T.D., Borg, C.K.H., Seshadri, R., Bonificio, W.D., and Clarke, D.R. (2013). Data-driven review of thermoelectric materials: performance and resource considerations. Chem. Mater. *25*, 2911–2920.

34. Bach, H., and Küppers, H. (1978). Cadmium diiodate. Acta Crystallogr. Sect. B *34*, 263–265.

35. Thévet, F., Dagron, C., and Flahaut, J. (1976). Contributionàl'étude du système formépar l'étain, le soufre et l'iode. Mise en évidence des deux variétés de l'iodosulfure stanneux Sn_2SI_2: comportement thermique et étude structurale. J. Solid State Chem. *18*, 175–182.

36. Chen, W.-T., Kuang, H.-M., and Chen, H.-L. (2010). Solid-state syntheses, crystal structures and properties of two novel metal sulfur chlorides $Zn_6S_5Cl_2$ and $Hg_3ZnS_2Cl_4$. J. Solid State Chem. *183*, 2411–2415.

37. Harding, J., and Pyper, N. (1995). The meaning of the oxygen second-electron affinity and oxide potential models. Philos. Mag. Lett. *71*, 113–121.

38. Oganov, A.R., Lyakhov, A.O., and Valle, M. (2011). How evolutionary crystal structure prediction works - and why. Acc. Chem. Res. *44*, 227–237.

39. Hautier, G., Fischer, C., Ehrlacher, V., Jain, A., and Ceder, G. (2011). Data mined ionic substitutions for the discovery of new compounds. Inorg. Chem. *50*, 656–663.

40. Heyd, J., and Scuseria, G. (2004). Efficient hybrid density functional calculations in solids: assessment of the Heyd Scuseria–Ernzerhof screened coulomb hybrid functional. J. Chem. Phys. *121*, 1187.

41. Heyd, J., Scuseria, G.E., and Ernzerhof, M. (2006). Erratum: "Hybrid functionals based on a screened Coulomb potential", [J. Chem. Phys. 118, 8207 (2003)]. J. Chem. Phys. *124*, 219906–1.

42. Wahila, M.J., Butler, K.T., Lebens-Higgins, Z.W., Hendon, C.H., Nandur, A.S., Treharne, R.E., Quackenbush, N.F., Sallis, S., Mason, K., Paik, H., et al. (2016). Lone-pair stabilization in transparent amorphous tin oxides: a potential route to p-type conduction pathways. Chem. Mater. *28*, 4706–4713.

43. Goldschmidt, V.M. (1937). The principles of distribution of chemical elements in minerals and rocks. The seventh Hugo Müller Lecture, delivered before the Chemical Society on March 17th, 1937. J. Chem. Soc. 655.

44. Shannon, R.D. (1976). Revised effective ionic radii and systematic studies of interatomic distances in halides and chalcogenides. Acta Crystallogr. Sect. A. *32*, 751–767.

45. Woodward, P.M. (1997). Octahedral tilting in perovskites. Geometrical considerations. Acta Crystallogr. Sect. B Struct. Sci. *53*, 32–43.

46. Hahn, H., and Mutschke, U. (1957). Untersuchungen ber ternre chalkogenide. xi. versuche zur darstellung von thioperowskiten. Z. Anorg. Allg. Chem. *288*, 269–278.

47. Lelieveld, R., and IJdo, D.J.W. (1980). Sulphides with the $GdFeO_3$ structure. Acta Crystallogr. Sect. B *36*, 2223–2226.

48. Nitta, T., Nagase, K., and Hayakawa, S. (1970). Formation, microstructure, and properties of barium zirconium sulfide ceramics. J. Am. Ceram. Soc. *53*, 601–604.

49. Sun, Y.-Y., Agiorgousis, M.L., Zhang, P., and Zhang, S. (2015). Chalcogenide perovskites for photovoltaics. Nano Lett. *15*, 581–585.

50. Bennett, J.W., Grinberg, I., and Rappe, A.M. (2009). Effect of substituting of s for o: the sulfide perovskite $BaZrS_3$ investigated with density functional theory. Phys. Rev. B *79*, 235115.

51. Goodenough, J.B., and Kafalas, J.A. (1973). Exploring the $A^{+}B^{5+}$O3 compounds. J. Solid State Chem. *6*, 493–501.

52. Castelli, I.E., Garca-Lastra, J.M., Hser, F., Thygesen, K.S., and Jacobsen, K.W. (2013). Stability and bandgaps of layered perovskites for one- and two-photon water splitting. New J. Phys. *15*, 105026.

53. Haynes, W.M. (2011). CRC Handbook, 92nd Edition (CRC Press).

54. J.S. Coursey, D.J. Schwab, J.J. Tsai. NIST Physical Measurement Laboratory, Standard Reference Database 144, http://www.nist.gov/pml/data/comp.cfm .

55. Cordero, B., Gómez, V., Platero-Prats, A.E., Revés, M., Echeverría, J., Cremades, E., Barragán, F., and Alvarez, S. (2008). Covalent radii revisited. Dalton Trans. 2832.

56. Andersen, T., Haugen, H.K., and Hotop, H. (1999). Binding energies in atomic negative ions: III. J. Phys. Chem. Ref. Data *28*, 1511.

57. Harrison, W.A. (1980). Electronic Structure and the Properties of Solids (W.H. Freeman).

58. Herman, F., and Skillman, S. (1963). Atomic Structure Calculations (Prentice Hall).

59. Kramida, A., Ralchenko, Yu., Reader, J., and NIST ASD Team. (2015). NIST Atomic Spectra Database (Ver. 5.3) (National Institute of Standards and Technology). http://physics.nist.gov/asd.

60. Pauling, L. (1960). The Nature of the Chemical Bond, Third Edition (Cornell University Press).

61. Allen, L.C., and Huheey, J.E. (1980). The definition of electronegativity and the chemistry of the noble gases. J. Inorg. Nucl. Chem. *42*, 1523–1524.

Chem, Volume *1*

Supplemental Information

Computational Screening of All Stoichiometric Inorganic Materials

Daniel W. Davies, Keith T. Butler, Adam J. Jackson, Andrew Morris, Jarvist M. Frost, Jonathan M. Skelton, and Aron Walsh

I. SUPPLEMENTAL DATA ITEMS

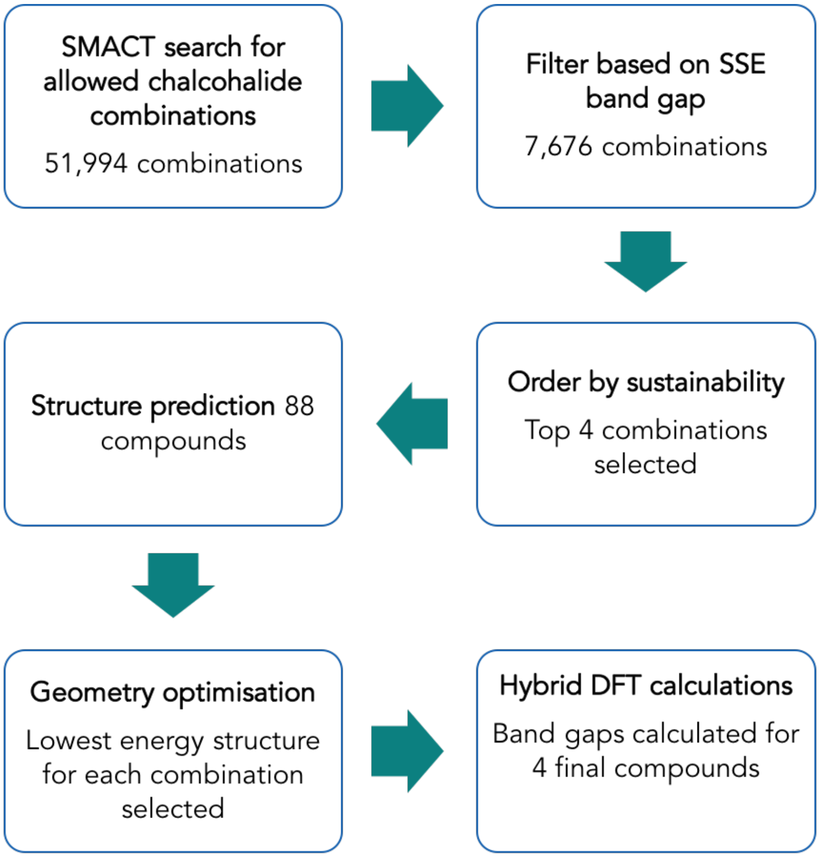

FIG. S1. Computational workflow: searching the combinatorial space for photoelectrochemical water splitting materials.

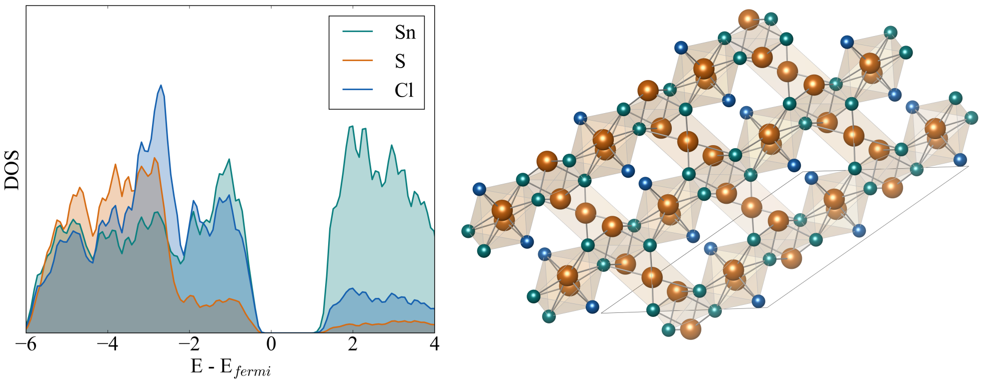

FIG. S2. (left) Electronic density of states and (right) predicted crystal structure of $Sn_5S_4Cl_2$.

TABLE S1: Comparison of measured bandgaps[1] (E_g^{exp}) against those predicted from the SSE model (E_g^{SSE}).

Material	E_g^{exp} (eV)	E_g^{SSE} (eV)
$MgSiP_2$	2.60	2.03
$ZnSiP_2$	1.70	2.00
$ZnSiAs_2$	1.00	1.93
$ZnGeN_2$	4.00	2.67
$ZnGeP_2$	1.70	2.14
$ZnGeAs_2$	1.00	1.15
$ZnSnP_2$	1.30	1.66
$ZnSnAs_2$	0.60	0.75
$ZnSnSb_2$	0.50	0.40
$CdSiP_2$	1.20	2.20
$CdSiAs_2$	0.50	1.55
$CdGeP_2$	1.20	1.73
$CdGeAs_2$	0.50	0.57
$CdSnP_2$	1.20	1.17
$CdSnAs_2$	0.50	0.26
$ZnGa_2S_4$	2.40	3.25
$ZnGa_2Se_4$	2.60	2.18
$ZnIn_2S_4$	1.80	2.87
$ZnIn_2Se_4$	2.00	1.68
$ZnIn_2Te_4$	1.40	1.35
$CdAl_2S_4$	1.90	3.40
$CdGa_2S_4$	1.90	3.16
$CdGa_2Se_4$	2.10	2.33
$CdGa_2Te_4$	1.50	1.50
$CdIn_2S_4$	1.80	2.21
$CdIn_2Se_4$	2.00	1.83
$CdIn_2Te_4$	1.40	1.15

$MgGa_2S_4$	2.50	3.40
$MgGa_2Se_4$	2.70	2.20
AsSBr	1.40	2.50
SbSI	1.50	1.88
SbSBr	1.50	2.26
SbSeBr	1.70	1.92
SbSeI	1.50	1.68
SbTeI	1.10	1.28

TABLE S2: Calculated bandgaps of top compounds identified by the screening procedure based upon density functional theory calculations (HSE06 functional) of the predicted crystal structures.

ABC combination	Formula	E_g^{calc} (eV)
CdSCl	$Cd_5S_4Cl_2$	2.96
CdSF	Cd_4SF_6	3.40
SnSCl	$Sn_5S_4Cl_2$	1.62
SnSF	Sn_4SF_6	3.00

II. SUPPLEMENTAL COMPUTATIONAL PROCEDURES

A. Validation of ternary bandgaps using the solid-state energy scale

The SSE dataset was initially built from binary compounds. In the original paper[2] the authors speculate about its applicability to ternary and higher order materials; however, we can find no reports of any such application. In order to assess whether the bandgap of a ternary material can be estimated from the difference between the highest anion and lowest cation SSE, we have tested this method against a set of well-characterised ternary semiconductor bandgaps.[1]. We compare to 35 materials, covering III-IV-V_2, II-III_2-VI_4 and V-VI-VII compounds, including metal halides, chalcogenides and pnictides. The agreement is reasonable, with a root-mean-squared deviation between of 0.66 eV. The data are presented in Table S1.

B. Workflow for selecting candidate photoelectrodes

The six step procedure that we adopt is shown schematically in Figure S1.

1. Allowed chalcohalide combinations

The constraints of charge neutrality and electronegativity are applied to all possible $A_xB_yC_z$ combinations with $B = [O, S, Se, Te]$ and $C = [F, Cl, Br, I]$. Stoichiometry is restricted to $A_xB_yC_z$, where the integers $w, x, y, z \leq 8$. Additionally we limit the A cations to those with an SSE higher than the water reduction potential (approx. -4.5 V relative to the vacuum at pH = 0). This results in 51,994 combinations.

2. SSE bandgap filter

The elemental combinations with a bandgap outside the range of 1.5 – 2.5 eV according to the SSE scale are discarded. Since $\sim$ 2 eV would represent an ideal bandgap, the $\pm$0.5 eV range allows sufficient space to allow for the uncertainty in the predicted SSE values. This results in 7,676 allowed combinations.

3. *Sustainability filter*

The sustainability of the 7,676 $A_xB_yC_z$ combinations is assessed based on sum the HHI_R scores of the three elements. The 20 combinations with the smallest HHI_R scores are shown in Figure 2 and the four combinations with the smallest HHI_R scores are taken forward to the structure prediction stage.

4. *Structure prediction*

In order to ascribe three-dimensional structures to the four element combinations, we use the approach developed by Hautier *et al.*[3] based on structural analogy. It suggests probable structure types based on the likelihood of ionic substitutions in existing compounds with known crystal structures. This procedure enables a rapid screening step which returns possible compounds with an associated probability of each crystal structure being adopted. We use a probability threshold of 0.001 and the Materials Project as the database for existing compounds. This results in a total of 88 structures to be taken forward to the density functional theory (DFT) optimisation step.

5. *Crystal structure optimisation*

For the structural relaxations, we employ DFT with a projector-augmented plane wave basis[4] and the PBEsol exchange-correlation functional[5] as implemented in the Vienna Ab-initio Simulation Package (VASP)[6,7]. A Monkhorst-Pack k-point grid was generated for each calculation with k-point spacing of 0.242 $Å^{-1}$. The kinetic energy cutoff is set at 500 eV and the force on each atom is converged to within 0.01 $eVÅ^{-1}$. For each of the four element combinations, the lowest total energy structure of those for which a local minimum could be found was taken forward to the bandgap calculation step.

6. *Electronic structure calculations*

Semi-local exchange-correlation treatments such as the PBEsol functional provide an accurate description of crystal structures but tend to underestimate the electronic bandgaps of semiconductors. To overcome this issue, computations of bandgaps are performed by

using the hybrid non-local functional HSE06,[8] which includes 25% screened Hartree-Fock exact exchange. The calculated bandgaps of the four final materials are presented in Table S2.

REFERENCES

[1]O. Madelung, *Semiconductors: Data Handbook* (Springer-Verlag, Berlin, Heidelberg, 2004).

[2]B. D. Pelatt, R. Ravichandran, J. F. Wager, and D. A. Keszler, J. Am. Chem. Soc. **133**, 16852 (2011).

[3]G. Hautier, C. Fischer, V. Ehrlacher, A. Jain, and G. Ceder, Inorg. Chem. **50**, 656 (2011).

[4]G. Kresse and D. Joubert, Phys. Rev. B **59**, 1758 (1999).

[5]J. P. Perdew, A. Ruzsinszky, G. I. Csonka, O. A. Vydrov, G. E. Scuseria, L. A. Constantin, X. Zhou, and K. Burke, Phys. Rev. Lett. **100**, 136406 (2008).

[6]G. Kresse and J. Furthmüller, Comput. Mater. Sci. **6**, 15 (1996).

[7]G. Kresse and J. Furthmüller, Phys. Rev. B **54**, 11169 (1996).

[8]A. V. Krukau, O. A. Vydrov, A. F. Izmaylov, and G. E. Scuseria, J. Chem. Phys. **125**, 224106 (2006).
